# Global Health care Professionals’ Perceptions of Large Language Model Use In Practice: Cross-Sectional Survey Study

**DOI:** 10.2196/58801

**Published:** 2025-05-12

**Authors:** Ecem Ozkan, Aysun Tekin, Mahmut Can Ozkan, Daniel Cabrera, Alexander Niven, Yue Dong

**Affiliations:** 1 Department of Medicine, Jersey Shore University Medical Center, 1945 NJ-33, Neptune, NJ, 07753, United States, 1 5078843064; 2 Department of Anesthesiology, Mayo Clinic College of Medicine, Rochester, MN, United States; 3 Department of Emergency Medicine, Mayo Clinic College of Medicine, Rochester, MN, United States; 4 Department of Pulmonary and Critical Care Medicine, Mayo Clinic College of Medicine, Rochester, MN, United States

**Keywords:** ChatGPT, LLM, global, health care professionals, large language model, language model, chatbot, AI, diagnostic accuracy, efficiency, treatment planning, patient outcome, patient care, survey, physicians, nurses, educators, patient communication, clinical, educational, utilization, artificial intelligence

## Abstract

**Background:**

ChatGPT is a large language model-based chatbot developed by OpenAI. ChatGPT has many potential applications to health care, including enhanced diagnostic accuracy and efficiency, improved treatment planning, and better patient outcomes. However, health care professionals’ perceptions of ChatGPT and similar artificial intelligence tools are not well known. Understanding these attitudes is important to inform the best approaches to exploring their use in medicine.

**Objective:**

Our aim was to evaluate the health care professionals’ awareness and perceptions regarding potential applications of ChatGPT in the medical field, including potential benefits and challenges of adoption.

**Methods:**

We designed a 33-question online survey that was distributed among health care professionals via targeted emails and professional Twitter and LinkedIn accounts. The survey included a range of questions to define respondents’ demographic characteristics, familiarity with ChatGPT, perceptions of this tool’s usefulness and reliability, and opinions on its potential to improve patient care, research, and education efforts.

**Results:**

One hundred and fifteen health care professionals from 21 countries responded to the survey, including physicians, nurses, researchers, and educators. Of these, 101 (87.8%) had heard of ChatGPT, mainly from peers, social media, and news, and 77 (76.2%) had used ChatGPT at least once. Participants found ChatGPT to be helpful for writing manuscripts (n=31, 45.6%), emails (n=25, 36.8%), and grants (n=12, 17.6%); accessing the latest research and evidence-based guidelines (n=21, 30.9%); providing suggestions on diagnosis or treatment (n=15, 22.1%); and improving patient communication (n=12, 17.6%). Respondents also felt that the ability of ChatGPT to access and summarize research articles (n=22, 46.8%), provide quick answers to clinical questions (n=15, 31.9%), and generate patient education materials (n=10, 21.3%) was helpful. However, there are concerns regarding the use of ChatGPT, for example, the accuracy of responses (n=14, 29.8%), limited applicability in specific practices (n=18, 38.3%), and legal and ethical considerations (n=6, 12.8%), mainly related to plagiarism or copyright violations. Participants stated that safety protocols such as data encryption (n=63, 62.4%) and access control (n=52, 51.5%) could assist in ensuring patient privacy and data security.

**Conclusions:**

Our findings show that ChatGPT use is widespread among health care professionals in daily clinical, research, and educational activities. The majority of our participants found ChatGPT to be useful; however, there are concerns about patient privacy, data security, and its legal and ethical issues as well as the accuracy of its information. Further studies are required to understand the impact of ChatGPT and other large language models on clinical, educational, and research outcomes, and the concerns regarding its use must be addressed systematically and through appropriate methods.

## Introduction

Large language model (LLM) refers to advanced artificial intelligence (AI) models designed for natural language processing tasks. LLMs are trained on vast amounts of text data and use deep learning techniques to understand and generate human-like language. They helped transform various fields, including medicine [1]. Some examples of most popular LLMs are LlaMA by Meta, Orca and Phi-1 by Microsoft, BLOOM, PaLM2 by Google, and GPT by OpenAI. ChatGPT, a chatbot powered by GPT-3/4 was released by OpenAI in November 2022, incorporating billions of parameters that enable it to comprehend and generate human-like text with the capability of context creation. Its intuitive interface and capacity for prompt engineering have enabled diverse applications across domains [2].

In medicine, recent studies have demonstrated ChatGPT’s potential to support clinical decision-making, summarize complex medical data, and streamline documentation processes. For instance, ChatGPT has been evaluated for its ability to generate discharge summaries, assist in developing differential diagnoses, and simplify patient communication [3-5]. Its role in medical education has also been explored, demonstrating its utility in preparing students for licensing exams like the United States Medical Licensing Examination (USMLE) and enhancing self-directed learning through case-based scenarios [5-7]. ChatGPT was also shown to be capable of defining and answering clinical vignettes and achieved >60% of the threshold on the USMLE, which is the passing score for all three exams [8,9]. Additionally, its ability to provide personalized health education and assist in chronic disease management has been highlighted as a promising avenue for improving patient outcomes [4,10].

The integration of ChatGPT into health care settings is accelerating, with a growing body of literature examining its applications. Despite these advancements, significant challenges remain. Concerns about data privacy, ethical implications, and the accuracy of AI-generated content persist as barriers to widespread adoption [4,5,10]. Additionally, little is known regarding global health care professionals’ perspectives and the extent and impact of ChatGPT’s integration in health care settings [11,12]. Most studies to date, have been limited to localized settings or specific subgroups. Yet, successful and ethical integration of ChatGPT into health care workflows depends heavily on end-user acceptance, awareness of limitations, and perceptions regarding safety, usability, and value [5-7].

This study aimed to evaluate health care professionals’ awareness and perceptions of ChatGPT, with a focus on its applications, challenges, and utility across clinical, educational, and research settings. We surveyed a diverse group of health care professionals—including physicians, nurses, researchers, and educators—from multiple countries and practice settings. Using a cross-sectional survey design, we collected data on their familiarity with ChatGPT, how and why they used it, and their concerns about its integration. Our a priori hypothesis was that while many health care professionals would recognize ChatGPT’s potential benefits, such as improving efficiency, communication, and access to knowledge, they would also express concerns regarding ethical, legal, and accuracy-related issues.

This study offers timely insights for health care leaders, educators, and policymakers considering the responsible adoption of generative AI tools. By reflecting on global perspectives from frontline users, our findings may help shape discussions on how to balance innovation with safety and trust in clinical AI applications.

## Methods

This study was conducted as a cross-sectional survey between April 20 and July 3, 2023 (Multimedia Appendix 1).

### Survey Instrument Development and Validation

The questionnaire used in this study was developed de novo by the research team. The design process was informed by the research team’s multidisciplinary experience in medicine, education, and digital health, as well as the evolving discourse around AI in health care. To assist with rapid prototyping, the research team used ChatGPT (OpenAI) to generate the first draft of the questionnaire. This initial draft provided a foundation for question phrasing and thematic organization. The final survey was iteratively refined by the study investigators to ensure clinical and contextual relevance.

To enhance clarity and assess feasibility, the questionnaire was piloted informally among five health care researchers affiliated with our institution. Their feedback informed improvements in question wording, branching logic, and estimated completion time (approximately 5 minutes). No formal psychometric validation was conducted.

The final survey included 33 questions and was distributed electronically using Research Electronic Data Capture (REDCap) (version 13.1.30; Vanderbilt University) [13]. The questionnaire was structured around six thematic domains: (1) respondent demographics and work environment, (2) awareness and familiarity with ChatGPT, (3) frequency and purpose of use, (4) perceived benefits and challenges of ChatGPT in daily practice, (5) views on ethical, legal, and data security concerns, and (6) future expectations and training needs. The questionnaire incorporated branching logic to adapt follow-up questions based on initial responses—for example, only respondents who reported using ChatGPT were asked about specific applications or frequency of use. A visual summary of the questionnaire flow and branching logic is provided in Multimedia Appendix 2. The final instrument has been reported in Multimedia Appendix 1.

### Participants and Sampling Strategy

We used a convenience sampling approach. The questionnaire was distributed to health care professionals via targeted emails, and professional Twitter, LinkedIn, and Instagram accounts using a snowball technique [14]. No predefined inclusion or exclusion criteria were applied beyond the requirement of being a health care professional (eg, physician, nurse, educator, researcher). There were no regional or institutional restrictions. As the survey was open and anonymous, we did not estimate a denominator or calculate a response rate. For the purposes of this study, we defined the application of ChatGPT in the medical field broadly to include its use in clinical care, research, medical education, and health care–related administrative tasks. This inclusive definition reflects the multifaceted roles that health care professionals fulfill and acknowledges that tools such as ChatGPT may support a wide range of activities beyond direct patient care, such as writing grants, academic correspondence, and synthesizing medical literature. Survey items were designed to capture this broad spectrum of use across domains relevant to daily professional practice.

Demographic information of participants was summarized. Among those familiar with ChatGPT, opinions on the tool and potential dissemination resources were assessed. For those who had not used it, barriers to usage were examined (Multimedia Appendix 2). Participants with experience using the ChatGPT were also asked about perceived challenges and approaches for enhancing usability. Summary statistics were provided as numbers and frequencies. Comparative analyses were conducted using the *χ*^2^ test, with a two-sided *P* value <.05 considered statistically significant. JMP Pro (version 14.1.0 software; SAS Institute Inc.) was used for the analyses.

### Ethical Considerations

The study protocol was evaluated by the Mayo Clinic institutional review board and it was determined that it was exempted under 45 CFR 46.102 of the Code of Federal Regulations (2/28/2023). No personally identifying information was collected, and all data were fully anonymous. Study participation was voluntary and survey completion was considered as consent. All survey responses were stored on secure, access-restricted servers in compliance with institutional data protection policies.

## Results

### Main Findings

**Figure 1.**
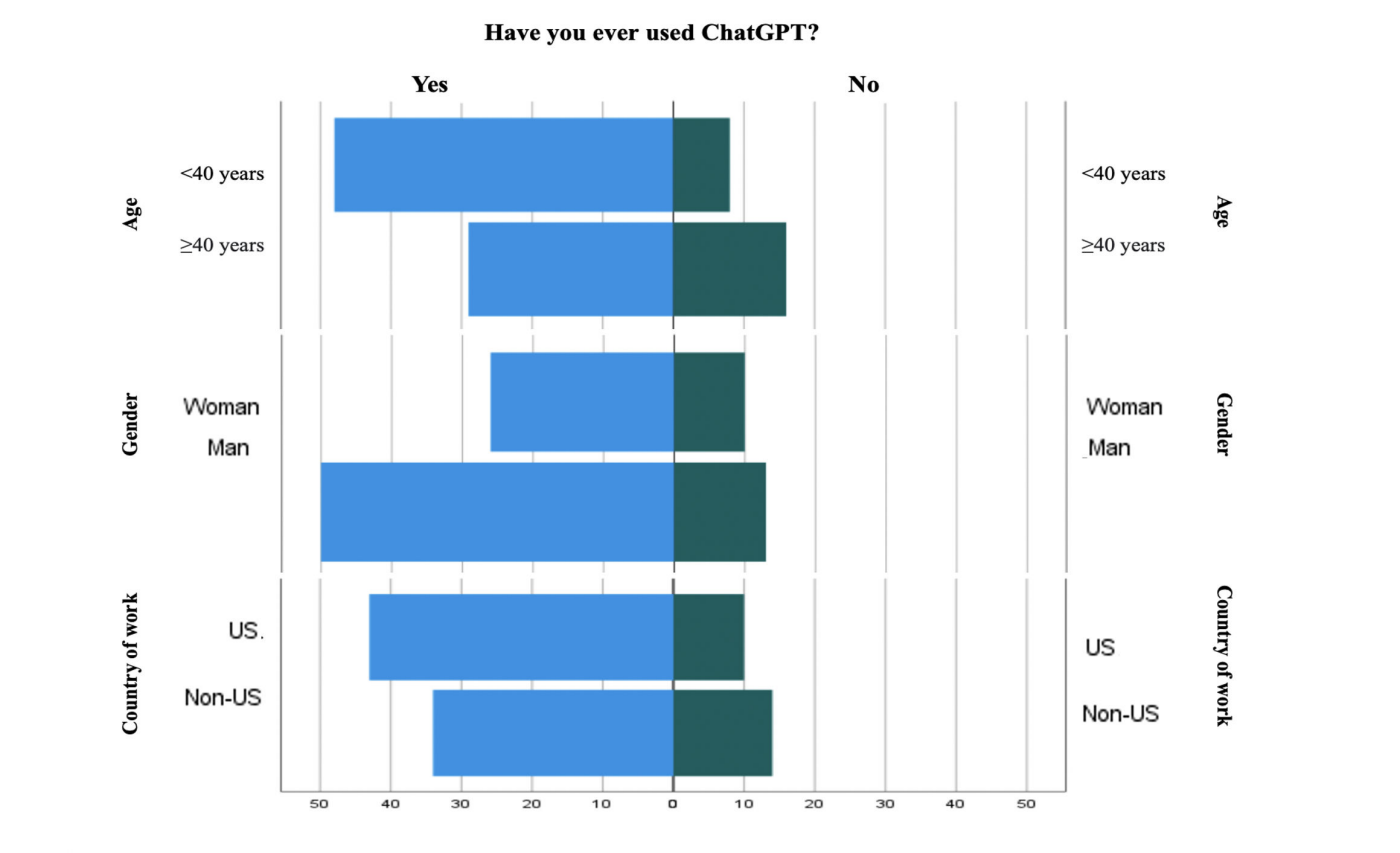
ChatGPT usage based on participants’ age, gender, and country of work.

A total of 115 health care professionals from 21 countries responded to the survey. Table 1 displays a summary of their demographic information (Figures1^2^).

**Table 1.** Baseline characteristics.

Variables	Participants (N=115), n (%)
Age (years)	
20‐29	30 (26.1)
30‐39	27 (23.5)
40‐49	26 (22.6)
50‐59	10 (8.7)
>60	22 (19.1)
Sex^a^	
Female	45 (39.5)
Male	68 (59.6)
Profession^a^	
Educator	16 (14.0)
NP/PA^b^	5 (4.4)
Physician	62 (54.4)
Researcher	25 (21.9)
RN^c^	5 (4.4)
Area/ Unit	
Internal medicine	20 (17.4)
Surgery	15 (13)
Emergency medicine	10 (8.7)
Psychiatry and Neurology	8 (7)
Anesthesiology/ICU^d^	10 (8.6)
Obstetrics and Gynecology	7 (6.1)
Radiology	6 (5.2)
Others^e^	39 (33.9)
Years since graduation	
<5	43 (37.4)
5‐10	27 (23.5)
11‐20	16 (13.9)
>20	29 (25.2)
Work length in hospital (years)^a^	
<5	66 (57.9)
5‐10	11 (9.6)
11‐20	19 (16.7)
>20	18 (15.8)
Country of work	
United States	53 (46.1)
Turkey	24 (20.9)
Tanzania	7 (6.1)
China	6 (5.2)
Croatia	3 (2.6)
Russia	2 (1.7)
France	2 (1.7)
Canada	2 (1.7)
Italy	2 (1.7)
Saudi Arabia	2 (1.7)
Others^e^	12 (10.4)
Native language	
English	28 (24.3)
Turkish	32 (27.8)
Spanish	10 (8.7)
Chinese (Mandarin)	9 (7.8)
Arabic	5 (4.3)
Others^e^	31 (26.8)
Place of employment^f^	
Academic hospitals and medical centers	72 (64.2)
Community hospitals	9 (8.0)
Private hospitals	13 (11.6)
Public hospitals	15 (13.4)
Free clinics	6 (5.4)
Others^e^	6 (5.3)
Frequency of ChatGPT usage (n=68)	
Multiple times per day	14 (20.6)
Once per day	3 (4.4)
Three to five times per week	14 (20.6)
Less than three times a week	13 (19.1)
Only tried it few times	24 (35.3)

a Due to lack of responses, missing data are not included in the reported totals; as a result, some category counts may not sum to the overall sample size.

b NP/PA: nurse practitioner/physician assistant.

c RN: registered nurse.

d ICU: intensive care unit.

e For Others see Multimedia Appendix 3.

f The subcategories are not mutually exclusive.

**Figure 2.**
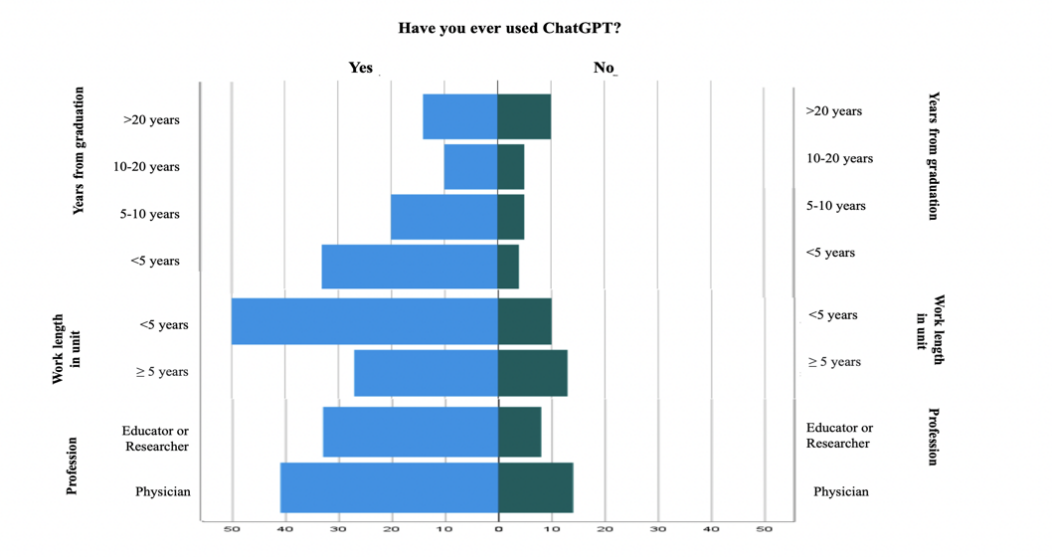
ChatGPT usage based on participants’ years since graduation, length of work in the current unit, and profession.

**Figure 3.**
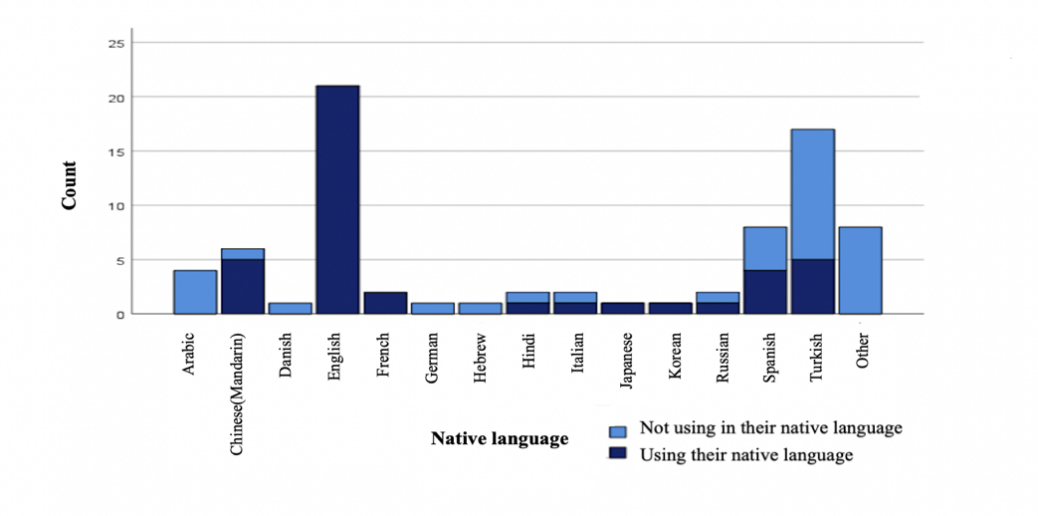
Ratio of native language use versus English use among participants while using ChatGPT.

**Figure 4.**
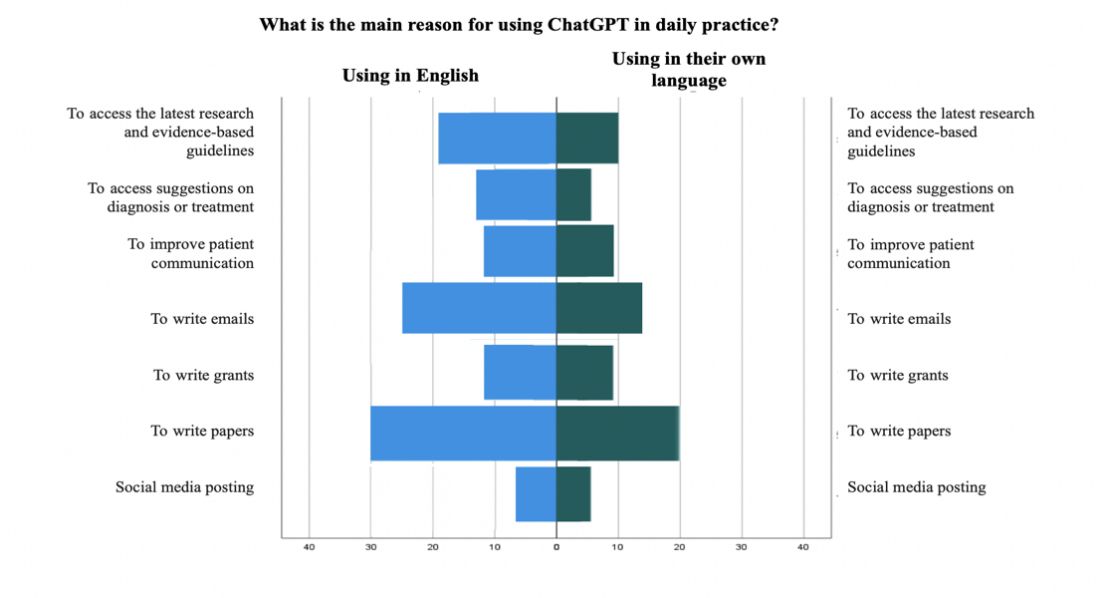
Main reasons for using ChatGPT in daily practice based on the language used by the participants.

Of the 115 participants, 101 (87.8%) had heard of ChatGPT, mainly from social media (n=33, 32.7%) and peers or colleagues (n=43, 42.6%). Of those, 77 (76.2%) had used ChatGPT before, with 18 (23.4%) using it multiple times per day and 23 (29.9%) having tried it only a few times. Moreover, 71 out of 77 (92.2%) participants used it in English. Among these, 50 were not native English speakers, and only 16/50 (32%) speakers used it both in English and their native language (Figure 3). Furthermore, variations in ChatGPT usage in daily practice were observed between participants using ChatGPT in English versus those who used it in their native language (Figure 4).

The most common reasons to use ChatGPT included writing papers (n=29, 44.6%) and emails (n=25, 38.5%), and obtaining suggestions on diagnosis or treatment (n=14, 21.5%) (Table 2). Additional reasons for ChatGPT usage by health care professionals in daily practice are shared in Table 3.

**Table 2.** ChatGPT usefulness based on used features in daily practice.

ChatGPT features	Participants (n=68), n (%)
Usefulness in daily practice
Not important	14 (20.6)
Slightly important	21 (30.9)
Moderately important	13 (19.1)
Important	13 (19.1)
Very important	7 (10.3)
ChatGPT’s usefulness, 0 (most negative experience) to 10 (most positive experience)	
≥7	42 (61.8)
4-5-6	19 (27.9)
≤3	7 (10.3)
Most useful features	
To access and summarize research articles efficiently	22 (46.8)
To provide quick answers to clinical questions	15 (31.9)
To provide patient education materials	10 21.3
To write emails, grants, and papers	25 53.2

**Table 3.** Percentage of participants’ main reasons for using ChatGPT in daily practice (multiple choice questions).

Main reason for using ChatGPT in daily practice	Participants (n=68), n (%)
Writing papers	31 (45.6)
Writing emails	25 (36.8)
To access the latest research and evidence-based guidelines	21 (30.9)
To access suggestions on diagnosis or treatment	15 (22.1)
To improve patient communication	12 (17.6)
To write grants	12 (17.6)

### Incorporation of ChatGPT Into Daily Practice

**Figure 5.**
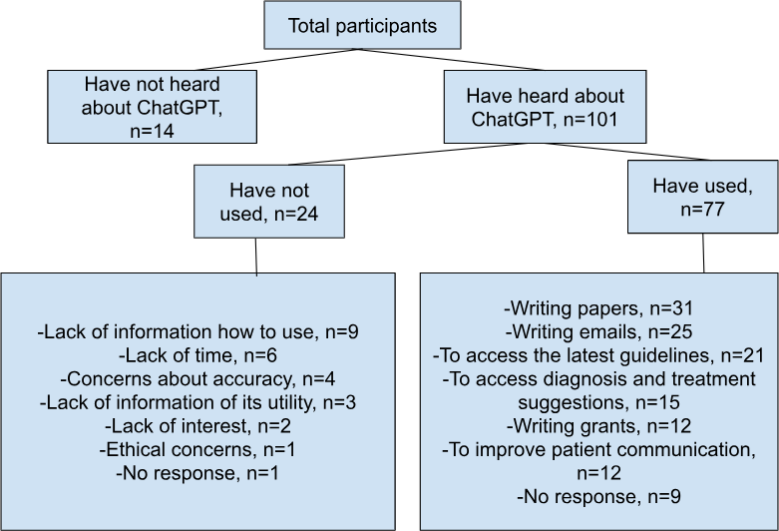
Factors contributing to use and nonuse of ChatGPT. The activities are not mutually exclusive and therefore, the total number of participants may exceed 115.

Of the 77 participants who used ChatGPT, 36 (46.8%) used ChatGPT in their clinical practice, 58 (75.3%) used it for research, and 56 out of 77 (72.7%) used it for educational activities (Figure 5).

Among all respondents, 42/101 (43.6%) participants agreed that they would not be concerned if their clinician used ChatGPT while providing care to them if they were the patient, whereas 32 (32.7%) disagreed and preferred that their clinician not use ChatGPT during care.

The majority (n=79, 78.2%) of participants agreed that ChatGPT could be useful for medical or health care professional education. In nonclinical settings, participants stated that ChatGPT could help to reduce workload (n=57, 73.1%), improve efficiency by automating certain tasks (n=51, 65.4%), offer greater access and efficiently summarize research articles (n=52, 66.7%), create patient educational materials (n=49, 62.8%), provide quick answers to questions (n=48, 61.5%), and enhance the ability to write papers (n=37, 47.4%).

### Challenges for Integrating ChatGPT Into Daily Practice

The main reasons for respondents not using ChatGPT included concerns about the accuracy of ChatGPT responses (n=14, 29.8%), limited applicability to their practice (n=18, 38.3%), legal and ethical considerations (n=6, 12.8%), limited diagnostic capabilities (n=4, 8.5%), lack of time (n=3, 6.4%), and lack of interest (n=2, 4.3%).

As one of the significant barriers is legal and ethical considerations, participants were asked to define plagiarism or copyright violations. Participants defined it as copying text or ideas from ChatGPT and using it for another source without citation (n=64, 63.4%), paraphrasing or summarizing content from ChatGPT and using it for another source without citation (n=41, 40.6%), using images from ChatGPT without permission (n=36, 35.6%), reusing or repurposing content from ChatGPT that was previously created for another purpose without permission (n=44, 43.6%).

In response to the legal and ethical challenges, participants proposed several solutions for integrating ChatGPT into daily practice. Participants stated that data encryption (n=63, 62.4%), access control (n=52, 51.5%), user authentication such as two-factor authentication (n=48, 47.5%), compliance with regulations such as Health Insurance Portability and Accountability Act or General Data Protection Regulation (n=62, 61.4%), transparency and informed consent (n=53, 52.5%), and regular training and awareness for health care professionals (n=58, 57.4%) are necessary to ensure patient privacy and data security.

### Views on ChatGPT’s success and other possible uses

When asked whether the participants knew ChatGPT had performed with ≥60% accuracy on the USMLE, 52 (51.5%) participants indicated they had heard this before. Additionally, 76 (68.5%) participants reported that they had not used any other AI platform.

Participants stated that ChatGPT can improve patient outcomes through personalized health education by providing tailored information and support (n=76, 75.2%); assisting with medication management through reminders and refill prescriptions, and provide information on side effects and interactions (n=55, 54.5%); telemedicine support for health care professionals to conduct virtual consultations, collect patient data, and provide decision support (n=48, 50%); aiding in symptom triage for patients (n=49, 48.5%); and offering mental health support by providing guidance on self-management techniques and coping strategies (n=49, 48.5%).

The distribution of responses based on different levels of postgraduate experience is reported in Multimedia Appendix 4. This distribution was largely balanced between the participants with fewer than 10 years and those with 10 or more years of experience.

## Discussion

### Principal Findings

This study offers a global perspective on how health care professional perceive and use ChatGPT in clinical, research, and educational context. Our findings demonstrate that awareness and adoption of ChatGPT are already widespread, with 76.2% of respondents having used the tool at least once. Participants primarily reported using ChatGPT for manuscript and email writing, grant application preparation, accessing research articles, clinical guideline support, diagnostic suggestions, and improving patient communication. Notably, more than three-quarters of participants agreed that ChatGPT holds potential utility in medical education, highlighting its ability to enhance learning experiences and facilitate task automation. Moreover, our study indicates that health care professionals endorse its use among colleagues. However, concerns about data privacy, ethical risks such as plagiarism, and the accuracy of AI-generated content remained as significant barriers to broader adoption. Proposed solutions included implementing safety protocols such as data encryption, access control, and regulatory compliance. In exploratory analyses comparing ChatGPT use, we did not identify significant differences across professional experience levels, which might be due to the limited sample size. Due to the wide range and uneven distribution of medical subspecialties represented, we were not able to conduct a formal comparison across specialties.

### Implications of Findings

Our findings highlight the broad and flexible potential of ChatGPT in health care workflows. In clinical practice, ChatGPT is perceived as a tool that can enhance efficiency by automating routine documentation tasks, such as generating draft discharge summaries and patient letters. It also supports decision-making by offering fast access to evidence summaries and aids communication through the creation of patient-friendly materials [5,15]. In medical education, participants identified ChatGPT as a valuable educational supplement—one that could be incorporated into curricula to simulate real-world clinical scenarios and assist in preparing students for standardized exams like the USMLE [5,16]. It can also support personalized learning experiences tailored to individual needs and self-directed learning pathways. In research, ChatGPT was valued for its ability in grant writing, literature synthesis, and ideation, especially in the early stages of manuscript development or protocol design [5].

These findings underscore the need for structured training programs and ethical guidelines to support responsible integration of AI tools. Implementing human-in-the-loop systems, in which clinicians oversee and validate AI outputs, may enhance safety, and build user confidence while mitigating risks associated with biases or inaccuracies in AI-generated content [17].

### Comparison to the Literature

Our findings align with prior studies that underscore ChatGPT’s potential in health care. Cascella et al [2] described ChatGPT’s potential to reduce administrative burden and assist with clinical reasoning, which mirrors participants’ reported use of ChatGPT for documentation and clinical queries. In medical education, Gilson et al [8] showed that ChatGPT achieved passing scores on all three components of the USMLE, highlighting its utility in medical education. Similarly, Kung et al [9] emphasized its role in creating standardized templates for patient education materials. These findings also align with our participants’ views on its usefulness for both learners and patients alike. Sallam [18] highlighted ChatGPT’s capacity to process and summarize complex medical data efficiently, which our participants also leveraged for research and evidence access.

However, our study adds unique insights by capturing global perspectives from diverse practice settings. Unlike prior reports focused on specific institutions or national populations, our results reflect a cross-disciplinary, international sample, offering a broader view of how generative AI is being perceived across diverse practice settings.

The main reasons behind the lack of use of ChatGPT in daily practice were mainly due to the nonapplicability to their practice, lack of information regarding its use, and concerns about the accuracy of ChatGPT’s responses, and legal and ethical considerations. The reason behind not using ChatGPT due to lack of information may be partially attributed to insufficient training opportunities for health care professionals in the use of generative AI. Previous studies have also indicated similar concerns regarding its implementation [19]. For instance, the concern for the spread of wrong information is a major obstacle, and different languages may have inconsistent results [20,21]. Many studies have shown that up to 96.7% of users are concerned about ethical and legal obstacles [3,18], particularly plagiarism [21-23], and copyright issues [3,18]. In a study conducted by a university at Sweden, 62% of students considered the use of chatbots for assignments and exams as cheating [24]. Our study showed that 86 out of 101 participants defined copying from ChatGPT as plagiarism. These concerns show that the implementation of ChatGPT into clinical settings will require a transition period supported by extensive safety measures. Health care professional leaders need to work with technology experts to develop learning objectives, curricula, assessments and evaluations, and safety protocols for this emerging technology.

Regarding the accuracy of ChatGPT’s responses, our study shows that health care professionals identified this as having a paramount importance. Similar studies have shown that ChatGPT should be used with caution due to potential biases of AI, which may lead to the generation of inaccurate information. When used in the health care system, this could potentially lead to harmful consequences [25].

### Educational Implications

The educational relevance of our findings is especially important. Our study suggests several opportunities:

- Curriculum design: Educators can incorporate ChatGPT into simulation- and case-based learning modules to foster clinical reasoning and application of evidence-based medicine.
- Needs Assessment: Educators may use baseline familiarity and usage patterns to tailor AI training initiatives and address gaps in knowledge or ethical understanding.
- Institutional Strategies: ChatGPT may serve as a tool in flipped classrooms, interactive tutorials, and self-directed learning, offering real-time feedback and access to guideline-driven responses.
- Learner Outcomes: By providing immediate feedback and access to evidence-based guidelines, ChatGPT has the potential to improve learner performance on standardized assessments [16].

Additionally, ChatGPT’s ability to generate accessible explanations for patients could enhance health literacy and improve communication between physicians and patients.

### Strengths and Limitations

This study has several strengths. We examined ChatGPT adoption from a global perspective. By including participants from 21 countries and various clinical and academic backgrounds, the study provides a valuable overview of current usage patterns and attitudes toward generative AI tools in health care. The survey instrument was comprehensive, capturing a wide range of use cases and concerns across clinical, research, and educational domains.

However, several limitations must be acknowledged. Although participants were from diverse countries, they are unlikely to represent the full range of health care professionals within their regions. The sample was likely skewed toward individuals with greater access to technology and academic networks, especially in countries where access to ChatGPT or certain social media platforms may be restricted or limited. Therefore, findings should be interpreted with caution and may not be generalized to all health care professionals in low-resource or digitally restricted settings. The use of convenience and snowball sampling likely introduced self-selection bias, attracting participants with preexisting interest in technology or AI. Because of this sampling method, we could not calculate a response rate. Most respondents were from academic hospital settings in the United States, which may limit applicability to other regions or practice environments. Conducting the survey in English may have limited the global inclusivity. Given the swift pace of technological advancements, particularly in generative AI applications such as ChatGPT and the continuous process of learning and integration by health care professionals, the present survey may not accurately capture the current perceptions and attitudes of doctors and nurses toward these technologies [26], limiting the temporal relevance of our findings . Lastly, although our survey included open-ended questions, multiple-choice questions may have led participants to an available answer.

### Future Directions

Further research is needed to address unanswered questions:

1. Long-term impact: Studies should evaluate how ChatGPT influences clinical outcomes, patient satisfaction, and educational performance over time.
2. Ethical frameworks: There is a pressing need for the development of institutional and regulatory guidelines governing AI use in health care [17].
3. Cross-language applications: Investigating how ChatGPT performs across different languages could help improve accessibility for non-English-speaking populations.
4. Training programs: Evidence-based strategies are needed to guide health care professionals in the ethical and effective use of generative AI technologies.

### Conclusion

ChatGPT usage is expanding within health care settings due to its variety of capabilities, and the majority of health care professionals are likely aware of its availability. It can improve the caliber of writing papers, grants, and emails; help health care professionals in accessing the latest guidelines, diagnosis, and treatment suggestions; and possibly improve patient communication. There are several concerns related to the implementation of LLMs in clinical practice, including legal, ethical, and operational issues. Further research is necessary to clarify the role of ChatGPT and LLM-based generative AI tools in health care education, research, and clinical practice.

## Supplementary material

10.2196/58801 Multimedia Appendix 1 ChatGPT Survey.

10.2196/58801 Multimedia Appendix 2 Diagram explaining survey flow.

10.2196/58801 Multimedia Appendix 3 Others within the demographic information table.

10.2196/58801 Multimedia Appendix 4 The distribution of answers to respondents with different levels of post-graduate experience.

## Abbreviations

AI: artificial intelligence
LLM: large language model
USMLE: United States Medical Licensing Examination
