# Supplementary material for: Global Health care Professionals’ Perceptions of Large Language Model Use In Practice: Cross-Sectional Survey Study
Source: JMIR Med Educ. 2025 May 12;11:e58801. doi: 10.2196/58801 (PMC12088617; doi:10.2196/58801)
Supplement: Multimedia Appendix 1 [file mededu-v11-e58801-s001.pdf]

Form 1

Record ID

____

What is your profession?

- ◯ Physician
- ◯ Nurse practitioner / Physician associate
- ◯ Nurse
- ◯ Pharmacist
- ◯ Respiratory therapist
- ◯ Other (please specify)

Other (please specify)

____

What is your area or unit?

- ◯ Allergy and Immunology
- ◯ Anesthesiology
- ◯ Colon and Rectal Surgery
- ◯ Dermatology
- ◯ Emergency Medicine
- ◯ Family Medicine
- ◯ Internal Medicine
- ◯ Medical Genetics and Genomics
- ◯ Neurological Surgery
- ◯ Nuclear Medicine
- ◯ Obstetrics and Gynecology
- ◯ Ophthalmology
- ◯ Orthopaedic Surgery
- ◯ Otolaryngology - Head and Neck Surgery
- ◯ Pathology
- ◯ Pediatrics
- ◯ Physical Medicine and Rehabilitation
- ◯ Plastic Surgery
- ◯ Preventive Medicine
- ◯ Psychiatry and Neurology
- ◯ Radiology
- ◯ Surgery
- ◯ Thoracic Surgery
- ◯ Urology
- ◯ Other

Other (please specify)

____

For how long have you been working since graduation from your most recent degree?

- ◯ < 5 years
- ◯ 5 to 10 years
- ◯ 10 to 20 years
- ◯ >20 years

For how long have you been working in your current hospital?

- ◯ < 5 years
- ◯ 5 to 10 years
- ◯ 10 to 20 years
- ◯ >20 years

In which country is your hospital located?

- ◯ Argentina
- ◯ Australia
- ◯ Austria
- ◯ Belgium
- ◯ Brazil
- ◯ Canada
- ◯ China
- ◯ Croatia
- ◯ Denmark
- ◯ Ecuador
- ◯ Finland
- ◯ France
- ◯ Germany
- ◯ Greece
- ◯ India
- ◯ Ireland
- ◯ Italy
- ◯ Japan
- ◯ Mexico
- ◯ Netherlands
- ◯ Norway
- ◯ Portugal
- ◯ Russia
- ◯ Serbia
- ◯ Slovenia
- ◯ Spain
- ◯ South Korea
- ◯ Sweden
- ◯ Turkey
- ◯ Ukraine
- ◯ United Kingdom
- ◯ United States
- ◯ Vietnam
- ◯ Other (please specify)

In which state do you work?

- ◯ Alabama
- ◯ Alaska
- ◯ Arizona
- ◯ Arkansas
- ◯ California
- ◯ Colorado
- ◯ Connecticut
- ◯ Delaware
- ◯ Florida
- ◯ Georgia
- ◯ Hawaii
- ◯ Idaho
- ◯ Illinois
- ◯ Indiana
- ◯ Iowa
- ◯ Kansas
- ◯ Kentucky
- ◯ Louisiana
- ◯ Maine
- ◯ Maryland
- ◯ Massachusetts
- ◯ Michigan
- ◯ Minnesota
- ◯ Mississippi
- ◯ Missouri
- ◯ Montana
- ◯ Nebraska
- ◯ Nevada
- ◯ New Hampshire
- ◯ New Jersey
- ◯ New Mexico
- ◯ New York
- ◯ North Carolina
- ◯ North Dakota
- ◯ Ohio
- ◯ Oklahoma
- ◯ Oregon
- ◯ Pennsylvania
- ◯ Rhode Island
- ◯ South Carolina
- ◯ South Dakota
- ◯ Tennessee
- ◯ Texas
- ◯ Utah
- ◯ Vermont
- ◯ Virginia
- ◯ Washington
- ◯ West Virginia
- ◯ Wisconsin
- ◯ Wyoming

Other (please specify) ________________________________

What is your native language?	◯ Arabic<br>◯ Chinese (Mandarin)<br>◯ Danish<br>◯ Dutch<br>◯ English<br>◯ Finnish<br>◯ French<br>◯ German<br>◯ Greek<br>◯ Hebrew<br>◯ Hindi<br>◯ Italian<br>◯ Japanese<br>◯ Korean<br>◯ Norwegian<br>◯ Portuguese<br>◯ Russian<br>◯ Spanish<br>◯ Swedish<br>◯ Vietnamese<br>◯ Turkish<br>◯ Other (Please specify):
Other (please specify)	__________________________________
How would you categorize your hospital?	◯ Academic Hospitals<br>◯ Community Hospitals<br>◯ Private Hospitals<br>◯ Public Hospitals<br>◯ Community Health Centers<br>◯ Free Clinics<br>◯ Retail Clinics<br>◯ Urgent Care Centers<br>◯ Other (please specify)
Other (please specify)	__________________________________
Age?	◯ 20-29<br>◯ 30-39<br>◯ 40-49<br>◯ 50-59<br>◯ 60 and above
Gender?	◯ Female<br>◯ Male<br>◯ Other<br>◯ Prefer not to disclose
Have you ever heard about ChatGPT?	◯ Yes<br>◯ No
Where did you first hear about ChatGPT?	◯ Social media<br>◯ Peers/Colleagues<br>◯ Google<br>◯ News/Newspaper<br>◯ Other (please specify)

Other (please specify)	______________________
Have you ever used ChatGPT?	◯ Yes<br>◯ No
If you have not used ChatGPT before, what was the main reason?	☐ Concerns about the accuracy of ChatGPT's responses<br>☐ Lack of time to use it<br>☐ Lack of interest<br>☐ Concerns about privacy and security<br>☐ Limited scope<br>☐ Prefer human interaction over technology<br>☐ None of the above<br>☐ Other (please specify):
What is the language you used for ChatGPT?	☐ Arabic<br>☐ Chinese (Mandarin)<br>☐ Danish<br>☐ Dutch<br>☐ English<br>☐ Finnish<br>☐ French<br>☐ German<br>☐ Greek<br>☐ Hebrew<br>☐ Hindi<br>☐ Italian<br>☐ Japanese<br>☐ Korean<br>☐ Norwegian<br>☐ Portuguese<br>☐ Russian<br>☐ Spanish<br>☐ Swedish<br>☐ Vietnamese<br>☐ Turkish<br>☐ Other (Please specify):
Other please specify	______________________
How frequently do you use ChatGPT?	◯ Multiple times per day<br>◯ Once per day<br>◯ Three to five times per week<br>◯ Less than three times a week<br>◯ I have only tried it few times
Have you ever used ChatGPT to search for something related to your daily clinical/research/educational practice?	◯ Yes<br>◯ No
How often do you use the ChatGPT in your daily clinical/research/educational practice?	◯ Multiple times per day<br>◯ Once per day<br>◯ Three to five times per week<br>◯ Less than three times a week<br>◯ I have only tried it few times

If you have used ChatGPT in your daily clinical/research/educational practice, what was the main reason?	☐ To access the latest research and evidence-based guidelines<br>☐ To access suggestions on diagnosis or treatment<br>☐ To write emails<br>☐ To write grants<br>☐ To write papers<br>☐ Social media posting<br>☐ None of the above<br>☐ Others (please specify):
Other (please specify)	__________________________________
If you have used ChatGPT in your daily clinical/research/educational practice, how would you rate your experiences? (0-10, 0: most negative experience, 10: most positive experience)	__________________________________
Could you please rate the usefulness of the ChatGPT in your unit's daily clinical/research/educational practice?	◯ Not important<br>◯ Slightly important<br>◯ Moderately important<br>◯ Important<br>◯ Very important
If you have not used ChatGPT in your daily clinical/research/educational practice, what was the main reason?	☐ Legal and ethical considerations<br>☐ Concerns about the accuracy of ChatGPT's responses<br>☐ Limited diagnostic capabilities<br>☐ Lack of time to use it<br>☐ Lack of interest<br>☐ Concerns about privacy and security<br>☐ Prefer human interaction over technology<br>☐ None of the above<br>☐ Other (please specify):
Other (please specify)	__________________________________
How do you think ChatGPT could be useful in your daily clinical/research/educational practice?	☐ Improving efficiency<br>☐ Enhancing patient care<br>☐ Reducing workload<br>☐ Providing additional resources for patient education<br>☐ Improving writing skills<br>☐ Improving statistical analysis<br>☐ None of the above<br>☐ Other (please specify):
Other (please specify)	__________________________________

If you were to use ChatGPT in your daily clinical/research/educational practice, what features would you find most useful?	☐ Ability to provide quick answers to clinical questions<br>☐ Ability to provide patient education materials<br>☐ Ability to access and summarize research articles efficiently<br>☐ Ability to provide diagnostic suggestions and treatment recommendations<br>☐ Ability to write emails, grants and papers<br>☐ Ability to analyze large amounts of medical data to identify patterns<br>☐ None of the above<br>☐ Other (please specify):
Other (please specify)	__________________________________
What other AI (AIGC) tools do you use?	☐ Artbreeder<br>☐ Copy.ai<br>☐ Colormind<br>☐ Craiyon<br>☐ DALL-E<br>☐ Designs.ai<br>☐ Elai<br>☐ Flexclip<br>☐ Frase IO<br>☐ Fronty AI<br>☐ Invideo<br>☐ Jasper<br>☐ Khroma<br>☐ Lovo.ai<br>☐ Lumen5<br>☐ Murf<br>☐ NightCafe<br>☐ Notion AI<br>☐ Peppertype<br>☐ Play.ht<br>☐ Replica<br>☐ Rytr<br>☐ Speechify<br>☐ starryai<br>☐ Synthesia<br>☐ Tome<br>☐ Uizard<br>☐ Veed.io<br>☐ None<br>☐ Other (please specify)
Other (please specify)	__________________________________
To what extent do you agree with the following statement:<br>If I were the patient, I would prefer my clinician to use ChatGPT while they are caring for me.	○ Strongly Disagree<br>○ Disagree<br>○ Neither agree or disagree<br>○ Agree<br>○ Strongly Agree
To what extent do you agree with the following statement:<br>ChatGPT can be useful for medical education.	○ Strongly Disagree<br>○ Disagree<br>○ Neither agree or disagree<br>○ Agree<br>○ Strongly Agree

How do you think ChatGPT could be useful in medical education?	☐ Ability to provide educational materials<br>☐ Ability to write papers<br>☐ Ability to provide quick answers to questions<br>☐ Ability to access and summarize research articles efficiently<br>☐ None of the above<br>☐ Other (please specify)
Other (please specify)	__________________________________
To what extent do you agree with the following statement:<br>According to my experience with ChatGPT, I would recommend the use of ChatGPT to other healthcare professionals.	◯ Strongly Disagree<br>◯ Disagree<br>◯ Neither agree or disagree<br>◯ Agree<br>◯ Strongly Agree
To what extent do you agree with the following statement:<br>AI technology can be used to generate original and high-quality papers without any instances of plagiarism.	◯ Strongly Disagree<br>◯ Disagree<br>◯ Neither agree or disagree<br>◯ Agree<br>◯ Strongly Agree
Did you know that ChatGPT performed ≥60% accuracy in USMLE?	◯ Yes<br>◯ No
