# Supplementary figures and images for: Global Health care Professionals’ Perceptions of Large Language Model Use In Practice: Cross-Sectional Survey Study
Source: JMIR Med Educ. 2025 May 12;11:e58801. doi: 10.2196/58801 (PMC12088617; doi:10.2196/58801)

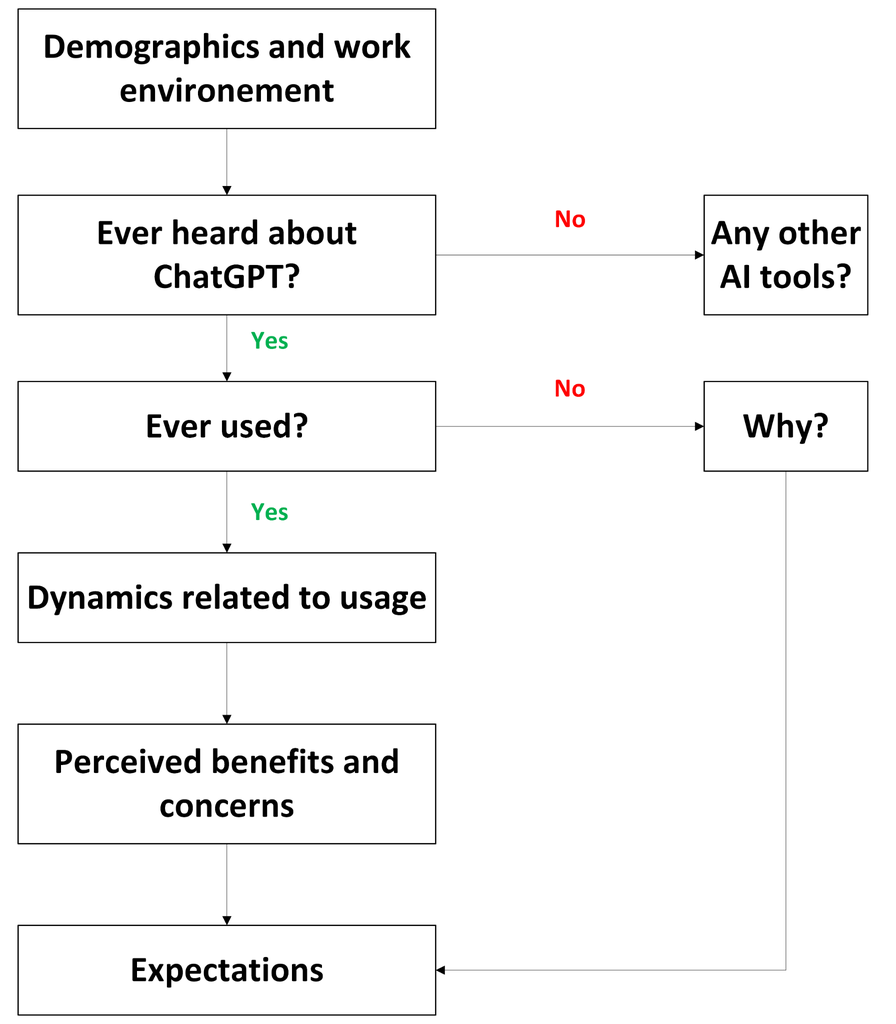

Supplement: Multimedia Appendix 2 [file mededu-v11-e58801-s002.png]
