# Supplementary material for: Global Health care Professionals’ Perceptions of Large Language Model Use In Practice: Cross-Sectional Survey Study
Source: JMIR Med Educ. 2025 May 12;11:e58801. doi: 10.2196/58801 (PMC12088617; doi:10.2196/58801)
Supplement: Multimedia Appendix 3 [file mededu-v11-e58801-s003.docx]

Area/Unit for who responded others
Dermatology (n=2), Family Medicine (n=3), Medical Genetics and Genomics (n=3), Ophthalmology (n=2), Pathology (n=2), Pediatrics (n=3), Physical Medicine and Rehabilitation (n=1), Urology (n=2), Microbiology (n=1), Simulation (n=1), Medical Surgical (n=2), Geriatrics (n=1), Health Information System (n=1), Public Health (n=1), Critical Care Medicine (n=1), Pain management (n=1), Research (n=4), Rheumatology (n=1), Cardiology (n=1), General Hospital Medicine (n=1), Physiology (n=2), Medical Education Central Administration (n=1), Medical Esthetic (n=1), Medical Laboratory (n=1), Unknown (n=1)

Country of work for who responded others
Argentina (n=1), Austria (n=1), Belgium (n=1), Brazil (n=1), Germany (n=1), Japan (n=1), Serbia (n=1), Vietnam(n=1)

Native language for who responded others
Swahili (n=3), Croatia (n=3), Russian (n=2), Hindi (n=2), Italian (n=2),French (n=2), Danish (n=2), Urdu (n=2), German (n=1), Hebrew (n=1), Japanese (n=1), Portuguese (n=1), Vietnamese(n=1), BCS (n=1), Farsi (n=1), African (n=1), Telugu (n=1), Maltese (n=1), Albanian (n=1), Serbian (n=1)

Type of employment for who responded others
Community Medical Center (n=1), Pharma Company (n=1), College of Nursing (n=1), Medical Laboratory (n=1), Retired (n=1)
