# Supplementary material for: Global Health care Professionals’ Perceptions of Large Language Model Use In Practice: Cross-Sectional Survey Study
Source: JMIR Med Educ. 2025 May 12;11:e58801. doi: 10.2196/58801 (PMC12088617; doi:10.2196/58801)
Supplement: Multimedia Appendix 4 [file mededu-v11-e58801-s004.docx]

|  |  | Years since graduation | | | |  |
| --- | --- | --- | --- | --- | --- | --- |
|  |  | **Less than ten years** | | **Ten or more years** | | **p value** |
|  | Have you ever used ChatGPT to search for something related to your daily clinical practice? | 24 | 45.3% | 12 | 50.0% | 0.701 |
|  | Have you ever used ChatGPT to search for something related to your daily research practice? | 40 | 75.5% | 18 | 75.0% | 0.965 |
|  | Have you ever used ChatGPT to search for something related to your daily educational practice? | 39 | 73.6% | 17 | 73.9% | 0.976 |
| Main reason for use | To access the latest research and evidence-based guidelines | 16 | 34.0% | 5 | 23.8% | 0.399 |
|  | To access suggestions on diagnosis or treatment | 8 | 17.0% | 7 | 33.3% | 0.204 |
|  | To improve patient communication | 6 | 12.8% | 6 | 28.6% | 0.168 |
|  | To write emails | 18 | 38.3% | 7 | 33.3% | 0.695 |
|  | To write grants | 7 | 14.9% | 5 | 23.8% | 0.493 |
|  | To write papers | 24 | 51.1% | 7 | 33.3% | 0.175 |
|  | Social media posting | 4 | 8.5% | 3 | 14.3% | 0.668 |
| Main reason for not use | Legal and ethical considerations | 5 | 15.6% | 1 | 6.7% | 0.648 |
|  | Concerns about the accuracy of ChatGPT's responses | 11 | 34.4% | 3 | 20.0% | 0.496 |
|  | Limited diagnostic capabilities | 3 | 9.4% | 1 | 6.7% | 1 |
|  | Lack of time to use it | 1 | 3.1% | 2 | 13.3% | 0.235 |
|  | Lack of interest | 1 | 3.1% | 1 | 6.7% | 0.541 |
|  | Lack of information about how to use it | 1 | 3.1% | 1 | 6.7% | 0.541 |
|  | Lack of information about its utility | 1 | 3.1% | 0 | 0.0% | 1 |
|  | Concerns about privacy and security | 3 | 9.4% | 0 | 0.0% | 0.541 |
|  | Prefer human interaction over technology | 2 | 6.3% | 2 | 13.3% | 0.583 |
|  | Limited scope | 5 | 15.6% | 0 | 0.0% | 0.162 |
| How often do you use the ChatGPT in your daily clinical/research/educational practice? | Multiple times per day | 11 | 23.4% | 3 | 14.3% | NA^*^ |
|  | Once per day | 2 | 4.3% | 1 | 4.8% |  |
|  | Three to five times per week | 8 | 17.0% | 6 | 28.6% |  |
|  | Less than three times a week | 8 | 17.0% | 5 | 23.8% |  |
|  | I have only tried it few times | 18 | 38.3% | 6 | 28.6% |  |
| Could you please rate the usefulness of the ChatGPT in your unit's daily clinical/research/educational practice? | Not important | 9 | 19.1% | 5 | 23.8% | NA* |
|  | Slightly important | 13 | 27.7% | 8 | 38.1% |  |
|  | Moderately important | 10 | 21.3% | 3 | 14.3% |  |
|  | Important | 11 | 23.4% | 2 | 9.5% |  |
|  | Very important | 4 | 8.5% | 3 | 14.3% |  |
| If you were to use ChatGPT in your daily clinical/research/educational practice, what features would you find most useful? | Ability to provide quick answers to clinical questions | 10 | 31.3% | 5 | 33.3% | 1 |
|  | Ability to provide patient education materials | 7 | 21.9% | 3 | 20.0% | 1 |
|  | Ability to access and summarize research articles efficiently | 15 | 46.9% | 7 | 46.7% | 0.989 |
|  | Ability to provide diagnostic suggestions | 4 | 12.5% | 2 | 13.3% | 1 |
|  | Ability to provide personalized and treatment recommendations | 3 | 9.4% | 5 | 33.3% | 0.089 |
|  | Ability to write emails, grants, and papers | 19 | 59.4% | 6 | 40.0% | 0.215 |
|  | Ability to analyze large amounts of medical data to identify patterns | 10 | 31.3% | 3 | 20.0% | 0.503 |
|  | Ability to improve efficiency by automating certain tasks, such as scheduling appointments and sending reminders to patients | 7 | 21.9% | 4 | 26.7% | 0.725 |
|  | Ability to monitor patients remotely by analyzing patient symptoms, and providing alerts when they require attention. | 3 | 9.4% | 2 | 13.3% | 0.648 |
|  | Ability to clarify EHR notes | 3 | 9.4% | 3 | 20.0% | 0.367 |
| What measures do you think would be necessary to ensure patient privacy and data security when using chatGPT? | Data encryption | 32 | 60.4% | 16 | 66.7% | 0.598 |
|  | Access control | 26 | 49.1% | 13 | 54.2% | 0.678 |
|  | User authentication such as two-factor authentication. | 24 | 45.3% | 13 | 54.2% | 0.47 |
|  | Compliance with regulations such as HIPAA or GDPR | 32 | 60.4% | 19 | 79.2% | 0.106 |
|  | Transparency and informed consent with the patient | 28 | 52.8% | 14 | 58.3% | 0.653 |
|  | Regular training and awareness for healthcare professionals | 29 | 54.7% | 17 | 70.8% | 0.182 |
